# R-MetaboList 2: A Flexible Tool for Metabolite Annotation from High-Resolution Data-Independent Acquisition Mass Spectrometry Analysis

**DOI:** 10.3390/metabo9090187

**Published:** 2019-09-17

**Authors:** Manuel D. Peris-Díaz, Shannon R. Sweeney, Olga Rodak, Enrique Sentandreu, Stefano Tiziani

**Affiliations:** 1 Department of Chemical Biology, Faculty of Biotechnology, University of Wrocław, J.Curie 14a, 50-383 Wrocław, Poland; manuel.perisdiaz@uwr.edu.pl; 2 Unidad Analítica, Instituto de Investigación Sanitaria La Fe (IIS La Fe), 46026 Valencia, Spain; 3 Dell Pediatric Research Institute (DPRI), University of Texas at Austin, Austin, TX 78723, USA; ssweeney@utexas.edu; 4 Institute for Cellular and Molecular Biology, The University of Texas at Austin, Austin, TX 78723, USA; 5 Department of Reproduction and Clinic of Farm Animals, Faculty of Veterinary Medicine, Wrocław University of Environmental and Life Sciences, 50-366 Wrocław, Poland; olga.rodak@upwr.edu.pl; 6 Instituto de Agroquímica y Tecnología de Alimentos (IATA-CSIC), Paterna, 46980 Valencia, Spain

**Keywords:** liquid chromatography high-resolution mass spectrometry, data-independent acquisition, all ion fragmentation, targeted analysis, untargeted analysis, metabolomics, R programming, full-scan MS/MS processing, R-MetaboList 2

## Abstract

Technological advancements have permitted the development of innovative multiplexing strategies for data independent acquisition (DIA) mass spectrometry (MS). Software solutions and extensive compound libraries facilitate the efficient analysis of MS^1^ data, regardless of the analytical platform. However, the development of comparable tools for DIA data analysis has significantly lagged. This research introduces an update to the former MetaboList R package and a workflow for full-scan MS^1^ and MS/MS DIA processing of metabolomic data from multiplexed liquid chromatography high-resolution mass spectrometry (LC-HRMS) experiments. When compared to the former version, new functions have been added to address isolated MS^1^ and MS/MS workflows, processing of MS/MS data from stepped collision energies, performance scoring of metabolite annotations, and batch job analysis were incorporated into the update. The flexibility and efficiency of this strategy were assessed through the study of the metabolite profiles of human urine, leukemia cell culture, and medium samples analyzed by either liquid chromatography quadrupole time-of-flight (q-TOF) or quadrupole orbital (q-Orbitrap) instruments. This open-source alternative was designed to promote global metabolomic strategies based on recursive retrospective research of multiplexed DIA analysis.

## 1. Introduction

Liquid chromatography high-resolution mass spectrometry (LC-HRMS) technology makes it feasible to simultaneously apply qualitative and quantitative approaches to the metabolite profiling of biological samples [1,2,3,4,5,6]. During the last decade, technological advances in electronics and hardware design have expanded multiplexing capacities, sensitivity and specificity of detectors, and facilitated the development of innovative scan options to address the needs of global metabolomics research [7]. Thus, traditional data-dependent acquisition (DDA), which requires the predetermined selection of precursors for MS/MS research, has been complemented by untargeted data-independent acquisition (DIA) approaches, such as all ion fragmentation (AIF) analysis [8,9,10]. This precursor-free strategy was initially introduced by Thermo Scientific in early Exactive benchtop Orbitraps for small-molecule applications in order to ameliorate the constraints of targeted analysis performed on triple-quadrupole (QQQ) detectors [9]. This operation mode was later adapted for modern hybrid quadrupole-Orbitrap (Thermo Q Exactive and Fusion Tribrid) and time-of-flight (q-TOF) detectors under different synonyms, such as all ion MS/MS, MS^ALL^, and MS^E^, depending on the manufacturer [11]. The flexibility of full-scan MS/MS analysis for targeted/untargeted-quantitative/qualitative research combined with high-throughput capacity of modern LC-HRMS detectors created a gap between hardware capabilities and licensed programs for in-depth automated processing of data from DIA analysis. While MS^1^ processing solutions are widely available and easily implementable [12,13,14], alternatives that address bulky AIF data processing are mainly limited to recently released open-source programs. This is the case for MS-DIAL, which was initially proposed for lipidomic research using a triple-TOF device [15], and later MetDIA, a solution with stated superior features for small molecule analysis while using the same detector [16]. Recently, the suitability of MS-DIAL for small molecule research assessed by quadrupole-Orbitrap AIF analysis has been shown [17]. However, the ability of these programs to reliably extract data from bulky DIA-MS files has not been demonstrated for small molecule research (*m/z* < 400). 

Recently, the R-package MetaboList was proposed as an accurate, flexible, and highly customizable alternative for full-scan MS/MS data processing [10]. The authors demonstrated the suitability of this approach for the study of metabolites with *m/z* < 250 while considering a mass tolerance of 5 ppm for both MS^1^ and MS/MS analyses collected by a quadrupole-Orbitrap detector. Interestingly, this study demonstrated how data analysis with R-MetaboList could be easily enhanced by continuous customization from users. From this, the suitability of R-MetaboList for small molecule research utilizing multiplexed full-scan MS-MS/MS experiments being performed on different LC-HRMS systems deserves further investigation. 

This research aims to demonstrate the flexibility and improved efficiency of an upgraded version of the previously released open-source R package MetaboList for metabolite research supported by LC full-scan MS^1^ and DIA-MS/MS analyses. A highly diluted human urine sample was analyzed in positive ionization mode by an LC-qTOF device that merged full-scan experiments at different collision-induced dissociation (CID) energies of 0, 5, 10, and 20 eV. Similarly, myeloid leukemia cells and medium extracts were studied by full-scan analyses on an LC q-Orbitrap system operating in fast polarity switching mode at 0% and 30% higher-energy collisional dissociation (HCD) energies. Automated processing of full-scan MS and MS/MS data for both HRMS instruments was carried out by R-Metabolist 2. Here, we demonstrate the utility of this data processing solution for the retrospective interrogation of DIA approaches to facilitate new insights for addressing global metabolomics of biological samples.

## 2. Results and Discussion

**Figure 1:**
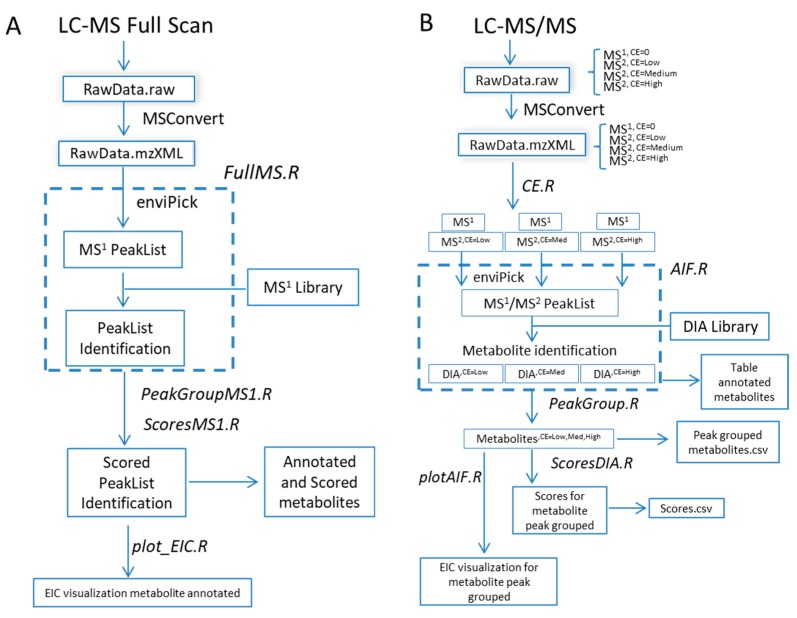
Overview of the R-MetaboList 2 workflow pipeline. (**A**) Initially, the raw data from an LC-MS full-scan experiment is converted to an .mzXML file format using MSConvert or other software. The file converted is processed by the *FullMS.R* function which performs a peak picking with the embedded enviPick algorithm to generate a peak list. A metabolite library consisting of neutral masses with optional retention time annotations is used by the *FullMS.R* function to provide a list of annotations that are grouped by metabolite assignment by the *PeakGroupMS1.R* function. Finally, the function *ScoresMS1.R* evaluates the isotope peak intensity ratio (IPIR), peak-to-peak Pearson correlation (PPC), and peak-to-peak shape (PPS) scores for each given metabolite. Finally, visualization of the extracted ion chromatogram (EIC) for the annotated metabolite is produced by the *plot_EIC.R* function. (**B**) Raw data from LC-MS/MS full-scan experiment is converted to an .mzXML file format which is further separated by collision energy (*CE.R*). MS^1^ at CE 0 and one MS^2^ per CE are processed by the *AIF.R* function, which performs a targeted extraction and putative annotation when an MS/MS library is provided. Peak grouping across CE values is performed with the *PeakGroup.R* function followed by scoring with the *ScoresDIA.R* function to evaluate the annotation confidence.

The R-MetaboList 2 package was developed in the R environment and it can be freely downloaded from the CRAN repository (https://CRAN.R-project.org/package=MetaboList) for automated targeted data extraction and the annotation of full-scan MS^1^ and/or MS/MS DIA spectra generated by LC-HRMS analysis. Figure 1 illustrates the workflow pipeline that was followed in this research and indicates the functions that are included in the R-MetaboList 2 package. In comparison with the previous version [10], the updated R-MetaboList 2 incorporates the following new features:

(1) Processing workflow for full-scan MS^1^ analysis (Figure 1A) independent of full-scan MS/MS analysis. The previous version did not include the processing of full-scan MS^1^ data outside the scope of the associated MS/MS data.
(2) Simultaneous processing of full-scan MS/MS data generated under different instrumental conditions (Figure 1B).
(3) Incorporation of scoring functions to evaluate metabolite annotation of both full-scan MS^1^ and MS/MS approaches (Figure 1B).
(4) Improved graphical representation of the results.
(5) Incorporation of a batch job function for compilation of full-scan MS^n^ reports from multiple samples for high-throughput applications.

### 2.1. Metabolite Profiling of Samples from Full-Scan MS^1^ Analysis

**Figure 2:**
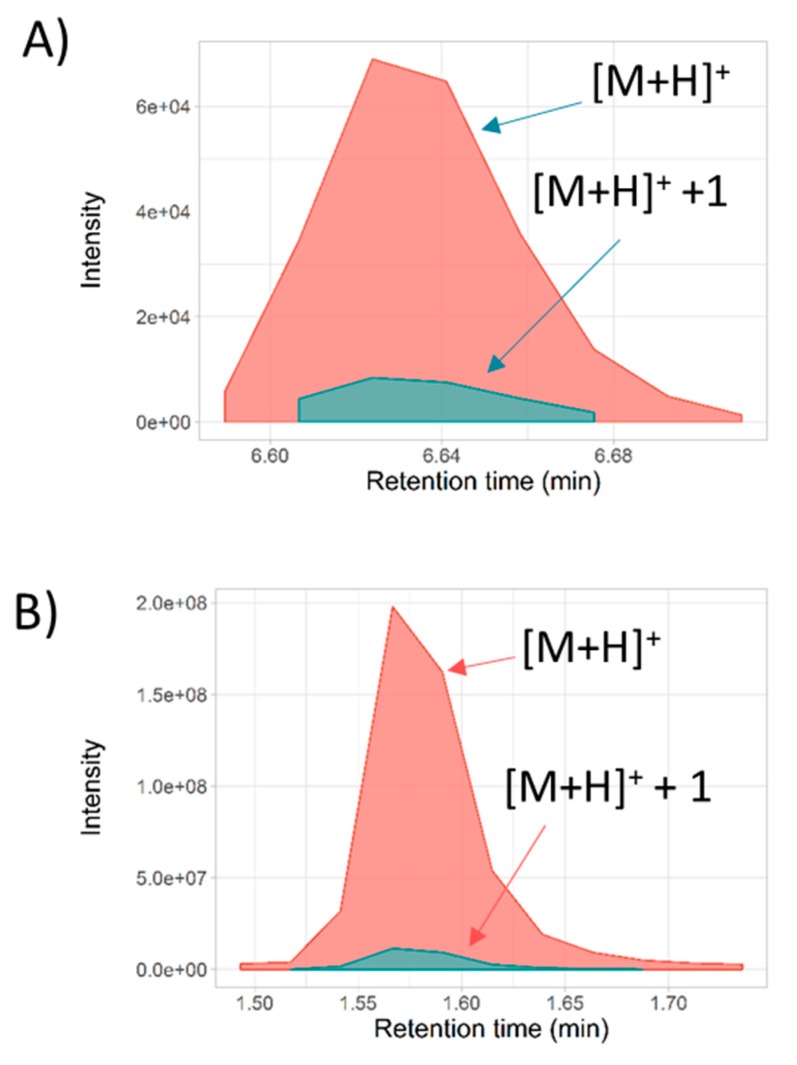
Graphical output generated by the *ScoresMS1.R* function. (**A**) Coelution extracted ion chromatograms (EIC) (extracted ion chromatogram) profile for phenylacetylglutamine detected in positive ionization mode with [M+H]^+^ and [M+H]^+^ +1 isotope for urine sample analyzed by LC-qTOF. (**B**) Coelution EIC profile for betaine detected in positive ionization mode with [M+H]^+^ and [M+H]^+^ +1 isotope putative identified in cell sample analyzed by LC-q-Exactive Orbitrap.

Metabolites annotation was initially addressed by R-MetaboList 2 through the processing of full-scan MS^1^ data from q-TOF and q-Orbitrap systems. Next, preliminary lists of tentative assignments that were generated by theoretical monoisotopic mass matching within a 5 ppm window were subsequently refined by full-scan MS/MS analysis. Peak picking of the underivatized urinary sample (q-TOF analysis) and targeted metabolite extraction by the *FullMS.R* function while considering the in-house neutral library utilized in this research (detailed in materials and methods section) yielded a total of 68 tentative metabolite assignments (Table S1). The tentative list of metabolites was grouped according to metabolite assignment and was exported in .csv file format (*PeakGroupMS1.R* function). Figure S1A,B illustrate the output style of the *plot_EIC.R* function (from *FullMS.R* function analysis) of [M+H]^+^ and [M+NH_4_]^+^ glutamine adducts in urine (qTOF), both annotated with less than 1 ppm mass deviation and a peak asymmetry factor of 1.5. The function (*plot_EIC.R*) produces a quality control plot that shows the *m/z* deviation for each scan forming the annotated peak (Figure S1C). Moreover, we designed a function named *ScoresMS1.R*, which incorporates the isotope peak intensity ratio (IPIR), peak-to-peak Pearson correlation (PPC), and peak-to-peak shape (PPS) scores (Detailed information in Section 3.3). Evaluation of the [M+H]^+^ and [M+NH_4_]^+^ glutamine adducts by the *ScoresMS1.R* function yielded a null PPC coefficient score revealing the absence of co-elution between both adducts. Similarly, urinary phenylacetylglutamine was detected in positive mode ([M+H]^+^) and the isotopic profile was resolved for the first isotopologue with a mass error lower than 5 ppm for both cases. The R package includes an IPIR score to increase the confidence of metabolite annotations. For metabolites with an absence of S or Br in the molecular formula, the IPIR should be greater than one. The extracted ion chromatogram (EIC) was plotted by the *plot_EIC.R* function and was evaluated by the *ScoresMS1.R* function which yielded a PPC score, IPIR, and asymmetry peak ratio of 0.99, 8.2, and 0.84, respectively (Figure 2A). 

Similarly, peak picking followed by the targeted feature extraction of cell and medium samples (q-Orbitrap analysis) led to 181 and 123 putative assignments, respectively (ESI, Tables S2 and S3 .csv). As an example of tentative assignments from cell and medium extracts using the q-Orbitrap instrument, betaine was found as [M+H]^+^ and its naturally occurring [M+H]^+^ +1 isotopologues in the cell sample with a mass accuracy below 1.5 ppm for both cases and peak asymmetry of 2.4 and 2.2, respectively (Figure 2B). Evaluation of both peaks by the *ScoresMS1.R* function resulted in a PPC score, IPIR, and asymmetry peak ratio of 0.99, 17, and 0.94, respectively. Overall, the workflow implemented for LC-MS full-scan analysis in the R-Metabolist 2 package generates a preliminary list of metabolites that can be confirmed by MS/MS analysis and/or retention time matching.

### 2.2. Metabolites Annotation by Full-Scan MS/MS Approach

Preliminary metabolite assignments that were achieved by the *FullMS.R* function in the urine sample analyzed by LC-qTOF were assessed by the *AIF.R* and *Filter_AIF.R* functions while using full-scan MS/MS data processing and loading the in-house MS/MS library detailed in Table S4A (positive ionization mode). Tentative MS/MS assignments were subsequently grouped by the *PeakGroup.R* function according to the appropriated CID assayed. Tables S5–S7 detail the tentative assignments achieved by peak grouping (alignment) of precursors and respective MS/MS fragments listed in Table S4 (positive ionization) at CID 5, 10, and 20 eV, respectively. Tentative assignments varied according to the CID assayed, although in all cases the mass error remained below 10 ppm and the retention time window for alignment was less than 0.1 min. Thus, 16, 20, and 23 metabolites were tentatively identified aligning the molecular mass with one (1 parent-fragment pair) of the respective fragment ions for CID 5, 10, and 20 eV, respectively. When considering alignment of molecular masses with their respective all fragment ions as a requisite for tentative assignments, there were annotated 11, 14, and 16 metabolites for CID 5, 10, and 20 eV, respectively. Election of the number of fragments that are required for tentative assignment was controlled by *Filter_AIF.R* functions embedded in R-MetaboList 2 and it can be customized by the user.

Data acquisition speed has a preponderant role in the sensitivity that is achieved by q-TOF analyzers, since higher scan rates decrease the accumulation time of ions. This is critical for low abundance species since high velocities can compromise detection. In contrast, scanning activity that is too slow permits the detection of minor compounds, but compromises the definition of the chromatographic response of all compounds (major and minor) by reducing the number of scans across each peak. An insufficient number of scans across any given peak results in increasing peak asymmetry, thus hindering quantitative analysis, as stated in a former version of R-MetaboList [10]. Moreover, there are numerous instrumental parameters that affect signal intensity, and thus optimization is required to increase the performance of MS detectors [18]. In this study, we focused on the suitability of the MS device for obtaining high-quality qualitative data without sacrificing quantitative analysis. An intermediate acquisition time of 250 ms was selected as a good compromise for multiplexed analyses (four scan events) of highly diluted samples. 

Moreover, it should be highlighted that the simultaneous calculation of signal intensities (Tables S5–S7) achieved at different collision energies (CE) greatly facilitates the election of appropriate breakdown energy according to the desired fragment being analyzed [19]. For example, the experimental glutamine peak group was formed by MS^1^ at 147.0764 *m/z* and two MS/MS fragments at 130.0499 and 84.0445 *m*/*z* (Table S8). At CE 5 and 10 eV both 130.0499 and 84.0445 *m/z* ([M+H]^+^) fragments were detected while at 20 eV only 84.0445 *m*/*z* fragment was found, revealing CE 5 and 10 eV as more suitable conditions for glutamine. Similarly, phenylacetylglutamine analyzed at CE 20 eV resulted in the absence of the 147.0763 *m/z* ion, whereas at CE 5 and 10 eV fragments at 84.0444, 130.0499, 136.0756, and 147.0763 *m/z* were observed (Table S8). On the other hand, for phenylalanine the optimum CID was found at CE 20 eV, in which both fragment ions, 103.0543 and 120.0808 *m/z*, appeared (Table S8). Overall, these results demonstrate minimal mass deviations and clearly indicate different optimal CID energies for maximized response of considered AIF fragments, depending on the molecule being studied (Table S5–S7).

Targeted metabolite extraction of full-scan MS/MS for cell extract analyzed by q-Orbitrap yielded a total of 53 and 51 tentative assignments (Table S9) when the alignment of molecular masses with one or all of their respective fragments (detailed in Table S4) was considered as an assignment constraint, respectively. Similarly, 29 and 26 metabolites were annotated in the medium sample (Table S10) when the alignment of molecular masses with one or all of their respective fragments, as listed in Table S4, is used as an assignment constraint.

The limited number of tentative assignments that were found in this study arose from the use of an early stage in-house AIF library listing 68 compounds and the analysis of highly diluted samples (mainly in the case of the urinary extract). More annotations can be achieved through the analysis of less diluted samples and/or the curation and use of a more extensive MS/MS AIF library that can be continuously expanded by users. In any case, these results demonstrate the flexibility of R-MetaboList 2 for processing multiplexed data generated by different LC-HRMS systems. The high-throughput capacity of such analytical platforms generates massive amounts of raw data that require the appropriate, customizable processing workflow to maximize the flexibility and reliability of biological data analysis. The manual handling of full-scan MS^1^ and MS/MS experiments is tedious and time-consuming. To ameliorate this problem, this research implemented a script (*AIF_Batch.R*, Supplementary Materials) that enables batch job processing of reports following parameter optimization. 

### 2.3. Selectivity for Metabolite Annotation by LC-DIA-MS: Quality Control and Scores Test

Once a full-scan MS^1^-MS/MS peak group is generated, further evaluation by statistical analyses can increase the confidence of the metabolite assignments. R-MetaboList 2 includes score tests based on the PPC score and PPS ratio for both quality control and product/precursor ion intensity ratios featured by the *ScoresDIA.R* function [20]. From our experience, a PPS value between 0.3 and 3 reflects acceptable similarity in chromatographic peak shape, however this parameter is defined by the discretion of the user. It should be noted that the PPC score is based on correlation coefficients and it can be overestimated when the EIC peaks are defined by an insufficient number of scans. It is recommended that 0.7 be set as the PPC cutoff for precursor-product scoring. To control potential overfitting, the function returns an intensity coelution plot of the scans shared by precursor/fragment peaks, as well as the correlation coefficient calculated by Pearson and *p*-value achieved by the fitting. The intensity co-elution plot also enables the inspection of the number of scans forming the peaks from precursor/fragment pairs. 

**Figure 3:**
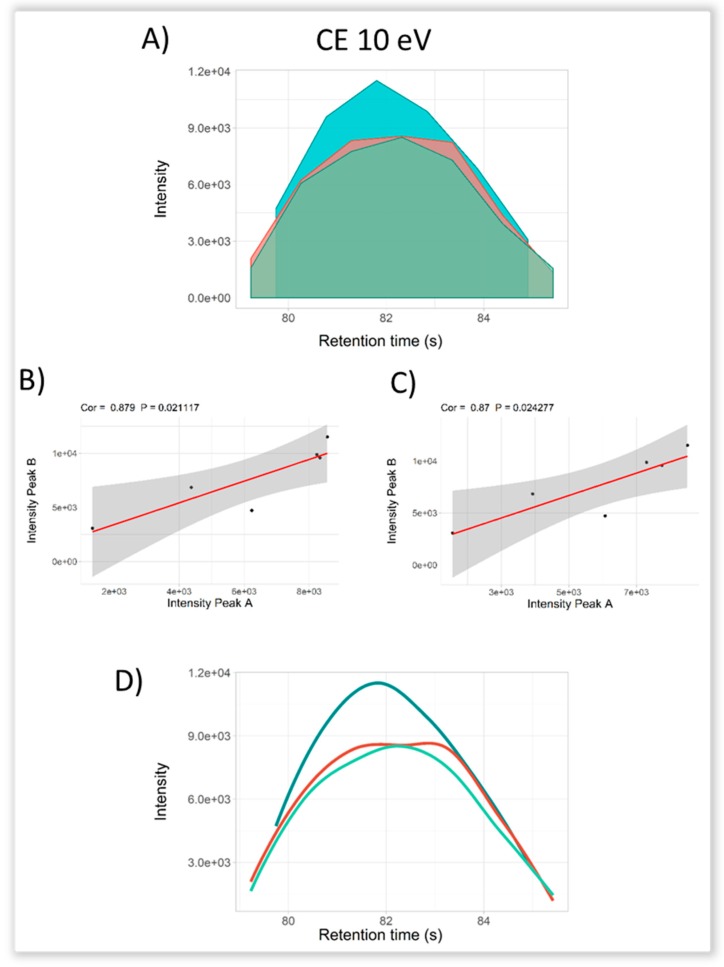
Peak visualization and statistical evaluation of glutamine characterized by LC-qTOF data independent acquisition (DIA)-MS/MS with the *ScoresDIA.R* function. Coelution profile for the EIC (extracted ion chromatogram) generated is plotted and followed by analysis of the peak-to-peak Pearson correlation (PPC) and peak-to-peak shape (PPS) ratio for the product/precursor ions. (**A**) Coelution profile for the precursor 147.0764 *m/z* and fragments 130.0499 *m/z* and 84.0444 *m/z* annotated as glutamine [M+H]^+^ obtained at 10 eV. (**B**) Peak-to-peak Pearson correlation analysis for 84.0444 *m/z* fragment with precursor ion. (**C**) Peak-to-peak Pearson correlation analysis for 130.0499 *m/z* fragment with precursor ion. (**D**). Smoothed coelution plot for PPC and PPS analysis.

Evaluating the feature previously annotated in urine as glutamine, scores were generated with the *ScoresDIA.R* function. The PPC was higher than 0.8 in all cases (Table 1). The fragment 84.0444 *m/z* that was obtained at CE 5 eV resulted in a PPS of 0.2 and a product/precursor ion intensity ratio of 0.2 and, thus, its annotation was not scored positively. However, this fragment was positively scored at CE 10 eV and 20 eV, in which the PPS was 0.67 for both and the product/precursor ion ratios were 0.74 and 0.99, respectively. Regarding the fragment 130.0499 *m/z*, at both CE 5 and 10 eV, all of the scores were satisfactory. Thus, we can conclude that glutamine analyzed at CE 10 eV produced fragment ion that can be most confidently annotated (Figure 3).

**Figure 4:**
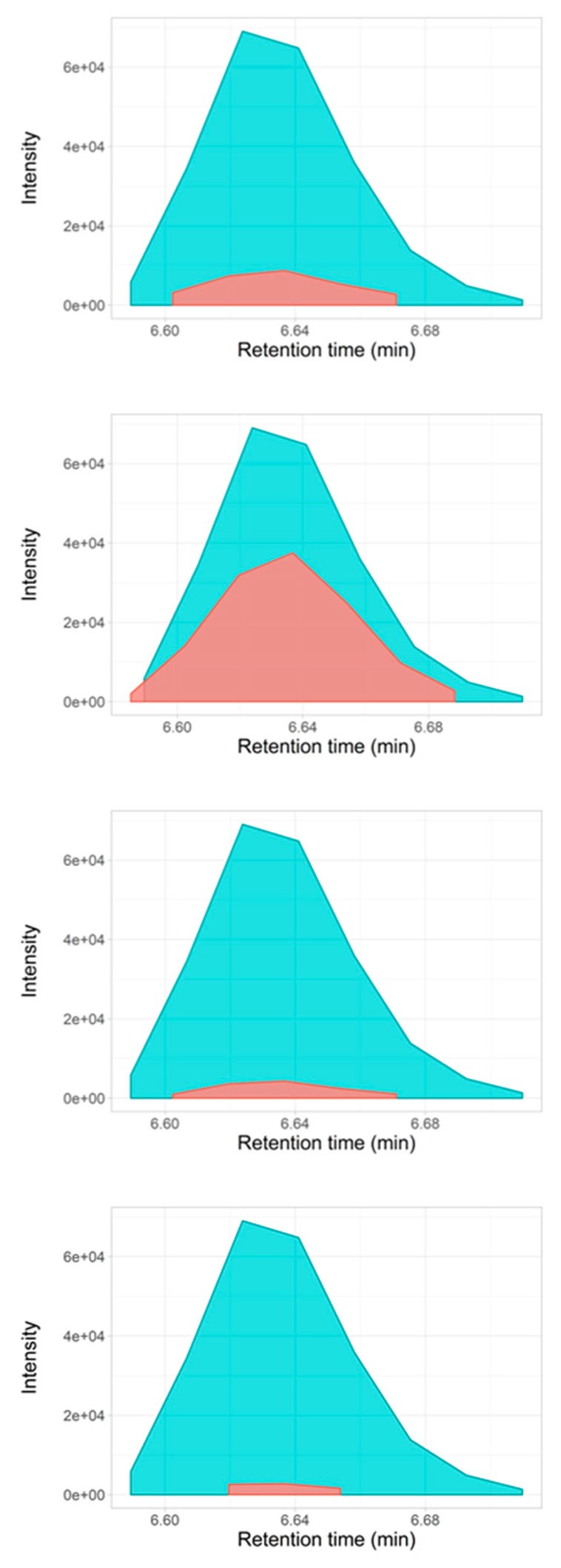
Extracted ion chromatograms (EIC) of phenylacetylglutamine precursor and fragment ions detected by LC-DIA-MS/MS at CE 20 eV generated with the *ScoresDIA.R* function. Figure shows coelution plots for each of the precursor-product pair ions from top to bottom: 84.0444 *m/z*, 130.0499 *m/z*, 136.0756 *m/z*, and 147.0763 *m/z*.

For phenylacetylglutamine, at CE 5 eV only 84.0444 and 130.0499 *m/z* fragments were found that coeluted with the respective precursor. However, the PPC score for the first fragment was too low and it was discarded as a positive annotation (Table 1). At CE 10 eV, a whole set of product ions were observed but with different PPC scores in comparison with their counterparts that were observed at CE 20 eV (Table 1). Product ion 136.0756 *m/z* [M+H]^+^ at CE 10 eV scored lower than the aforementioned recommended cutoff of 0.7, making the product-precursor association unreliable. As observed, CE 20 eV yielded the best results. Figure 4 shows the co-elution plot for the phenylacetylglutamine precursor and the product ions at 20 eV.

Regarding phenylalanine, the precursor ion at 166.0862 *m/z* was grouped with the AIF ion at 120.0809 *m/z* at the three CE voltages assayed and the scores were evaluated. The fragment ion that was obtained at CE 5 eV did not score above the cutoff threshold for PPC and scored in the upper limit for PPS, in contrast to CE 10 and 20 eV, which showed scores within the recommended values (Table 1). Moreover, the fragment ion 103.0543 *m/z* was also detected at CE 20 eV. 

To illustrate the results that were achieved by the q-Orbitrap approach, glutathione found in cell sample was statistically evaluated by the *ScoresDIA.R* function after its annotation with the *AIF.R* function. As observed in Table S9, all of the product ions were detected and coeluted with the [M+H]^+^ precursor ion 308.0903 *m/z*. In all cases, the PPC and PPS scores were within the cut-off thresholds (Table 2). Another scoring example was performed for methionine, which showed positive scoring except for the 133.0315 *m/z* fragment, which exhibited a PPS lower than 0.2 (Table 2). As previously commented, an insufficient number of scans across metabolite peaks can result in overestimated PPC scores, but also the opposite effect for PPS and, from this, the visualization of co-eluted precursor/fragments peaks is highly recommended. Extracted ion chromatograms (Figure S2) of the precursor (blue line) and the fragment 133.0315 *m/z* (red line) demonstrate co-elution and, thus, the low PPS scored is due to the low intensity of the fragment and number of scans per peak. Tyrosine is an example in which in all cases scores were within the recommended thresholds, indicating optimal parameters for the detection, fragmentation, and annotation of this metabolite (Table 2).

## 3. Materials and Methods

### 3.1. Chemicals and Sample Preparation

LC-MS grade methanol (MeOH), formic acid (FA), and acetonitrile (ACN) were from Fisher Scientific (Pittsburgh, PA, USA). Water was of ultrapure grade (EMD Millipore Co., Billerica, MA, USA). Two different batches of deuterated internal standards were prepared to be spiked as internal standards (IS) in samples that were separately studied by the approaches considered. Stable isotope-labeled D5-glutamic acid and D5-phenylalanine constituted the q-TOF IS mix. The q-Orbitrap IS mix contained D2-Fumaric acid, D3-DL-Glutamic acid, D3-Malic acid, D4-Citric acid, D4-succinic acid, D2-Cysteine, D5-Glutamine, D3-Serine, D3-Aspartic acid, and D5-L-Tryptophan. Labelled standards were purchased from Cambridge Isotope (Cambridge Isotope Laboratories Inc., Tewksbury, MA, USA). Deuterated standards were in the 98–99% and 97–99% chemical and isotopologue purity ranges, respectively. Internal standards were dissolved in 0.2% FA, diluted to a final concentration of 2 ppm, and the aliquots were kept at −80 °C until analysis. Commercial negative/positive calibration and reference (lock masses) solutions specific for the q-TOF device were purchased from Agilent (Agilent Technologies, Santa Clara, CA, USA). Positive and negative calibration solutions for the q-Orbitrap detector were from Thermo Scientific (Thermo Sci., San Jose, CA, USA). 

The underivatized 24-hour urine sample assayed was from a healthy human volunteer. It was centrifuged at 22,000 *g* at 4 °C for 15 min. An aliquot of the supernatant was diluted 1:1000 with ultrapure water, spiked with the q-TOF IS mix (final IS concentration in sample was 0.2 ppm), and filtered through a 0.2 µm nylon membrane. Aliquots of 150 µL were transferred to LC-MS vials and stored at −80 °C until analysis.

The cell and medium samples were prepared from acute myeloid leukemia cells (MOLM-13) cultured in RPMI-1640 medium supplemented with 10% characterized fetal bovine serum (FBS) and 2 mM L-glutamine (GE Healthcare Biosciences, Pittsburgh, PA, USA). Cells were incubated under standard conditions at 37 °C with 5% CO_2_ and maintained at a concentration range of 200,000 to 2 × 10^6^ cells/mL. Medium and cells were collected following a 24-hour incubation period. Suspension cells and medium were aspirated and centrifuged. Supernatant (conditioned medium) was snap frozen in liquid nitrogen. Prior to LC-MS analysis, medium was thawed, ultrafiltered (Nanosep centrifugal devices with Omega membrane, Pall Corporation, Port Washington, New York, USA), diluted 1:500 with ultrapure water, and then spiked with the q-Orbitrap IS mix (final IS concentration in sample was 0.2 ppm). Cells were washed twice with phosphate buffered solution (GE Healthcare Biosciences), harvested by centrifugation, and snap frozen in liquid nitrogen. Metabolite extraction was performed by modified Bligh-Dyer, as previously reported [10]. In brief, cell pellets were extracted with 1:1 water:methanol and equal parts chloroform. Following mixing and centrifugation, the polar fraction was transferred to Eppendorf tubes and then dried at 4 °C (Vacuum Concentrator, LabConco Corporation, Kansas City, MO, USA). Metabolites were resuspended in ultrapure water containing the q-Orbitrap IS mix (final IS concentration in sample was 0.2 ppm) and ultrafiltered before being transferred into LC-MS vials.

### 3.2. LC-MS/MS Analysis

Chromatographic separation of the underivatized urine sample was carried out on an Agilent 1290 Infinity II (Agilent Technologies, Santa Clara, CA, USA) HPLC system that was equipped with a quaternary pump, vacuum degasser, and an autosampler with a temperature controller coupled to an Agilent 6550 q-TOF mass analyzer equipped with an electrospray ionization (ESI) source with Jet Stream Technology. Metabolite separation was achieved on a 150 mm × 2.1 mm, 4 µm particle size Synergi-Hydro C18 column (Phenomenex Inc., Torrance, CA, USA) under the following separation conditions: solvent A, water/FA (99.8:0.2 v:v); solvent B, ACN; separation gradient, initially 1% B, held for 2 min., and then linear 1–80% B in 8 min., washing with 98% B for 2 min., and column equilibration with 1% B for 7 min.; total run time, 19 min.; flow rate, 0.25 mL/min; injection volume, 5 µL. Autosampler and column temperatures were set at 6 °C and 23 °C, respectively. Column flow was directed into the mass analyzer in the time range of 0.7–12 min., diverting the rest of the run time to waste. The samples were analyzed in positive ionization conditions operating in high-resolution full-scan MS mode with the settings: gas temperature, 130 °C; drying gas, 14 L/min.; nebulizer, 30 psig; sheath gas, 10 L/min.; isolation width, narrow (1.3 *m/z*); nozzle voltage, 500 V; fragmentor, 380 V; octapole 1 RF, 400V; capillary voltage, 3500 V; lock masses, 121.0509 *m/z* and 922.0098 *m/z*; data acquisition, centroid mode. Injections merged four full-MS analyses with CID collision energies of 0, 5, 10, and 20 eV with an acquisition rate of four spectra/s and 250 ms/spectrum as accumulation time. Polarity switching was not considered in this research because the mass deviations achieved by the MS device used were above 100 ppm regarding molecules at *m/z* <250. Before analysis, the MS device was tuned and calibrated in the low mass range and high-resolution mode (4 GHz) to maximize the mass accuracy of detection (considered mass tolerance was 10 ppm at all times). Additionally, the peak area ratio of D5-glutamic/D5-phenylalanine in the sample analyzed were compared to that observed in an aqueous model solution of IS at 0.2 ppm to confirm the absence of significant matrix effects.

The analysis of cell and media extracts was performed on a Thermo Accela HPLC system equipped with a quaternary pump, vacuum degasser, and an open autosampler with a temperature controller (Thermo Scientific, San José, CA, USA). Chromatographic separation of metabolites was achieved by the same reverse phase column described above with the following separation conditions: solvent A, water/FA (99.8:0.2); solvent B, MeOH; separation gradient, initially 5% B, held for two minutes and then linear 30–80% B in eight minutes, washing with 98% B for 10 min and column equilibration with 5% B for 10 min; flow rate, 0.25 mL/min.; injection volume, 5 µL; total run time, 30 min.; autosampler and column temperatures were set at 6 °C and 22 °C, respectively. Column flow was directed into the mass analyzer in the time range of 1–15 min. and diverted to waste outside this period. Mass spectrometry analysis was carried out on a Thermo Q Exactive Hybrid Quadrupole-Orbitrap benchtop detector that was equipped with an electrospray (ESI) source simultaneously operating in fast positive/negative polarity switching mode (Thermo Scientific, Bremen, Germany). Multiplexed full-scan MS^1^ (full-MS) and MS/MS (AIF) experiments had the following settings: microscans, 1; AGC target, 1e^6^; maximum injection time, 100 ms; mass resolution, 35,000 FWHM at *m/z* 200 for full-MS analysis whereas AIF scan conditions were microscans, 1; AGC target, 3e^6^; maximum injection time, 1000 ms; mass resolution, 70,000 FWHM at *m/z* 200; HCD energy, 30. In both cases, the instrument was set to spray voltage, 4.0 kV; capillary temperature, 300 °C; sheath gas, 55 (arbitrary units); auxiliary gas, 30 (arbitrary units); *m/z* range, 50–750; data acquisition, centroid mode. The accuracy of Orbitrap analysis was ensured by calibrating the detector while using the commercial calibration solutions that were provided by the manufacturer, followed by a customized adjustment for small molecular masses. Masses at *m/z* 87.00877 (Pyruvic acid); 117.01624 (D2-Fumaric acid); 149.06471 (D3-Glutamic acid); 265.14790 (Sodium dodecyl sulfate); and, 514.288441 (Sodium taurocholate) were used for the negative ionization mode, whereas masses at *m/z* 74.09643 (n-Butylamine), 138.06619 (Caffeine fragment), 195.08765 (Caffeine), and 524.26496 (Met-Arg-Phe-Ala tetrapeptide, MRFA) were used to adjust the mass accuracy of the positive ionization mode. Maximal mass tolerance was 5 ppm at all times. The LC-MS platform of analysis was controlled by a PC operating the Xcalibur v. 2.2 SP1.48 software package (Thermo Scientific, San Jose, CA, USA). Again, the ratios among spiked IS in samples and in an aqueous model solution at same concentration confirmed the absence of matrix effects.

### 3.3. Automated Data Processing by R-MetaboList 2

Agilent and Thermo experimental data files (extension .d and .raw, respectively) were converted into .mzXML files by the MSconvert option embedded in the freely available Proteowizard application (http://proteowizard.sourceforge.net/). Full-scan MS^1^ and MS/MS data were separated according to CID (0, 5, 10, and 20 eV for q-TOF) and HCD (0 and 30% for q-Orbitrap), and simultaneously assayed while using the *CE.isolation.R* function included in R-MetaboList 2. Peak picking of MS^1^ and MS/MS data was performed in the background by the enviPick algorithm embedded in the software in a stepped process [21] (Figure 1).

A preliminary full-MS (intact molecule) analysis of samples was carried out by R-MetaboList 2 loading an in-house neutral mass library (.csv format) of 320 underivatized metabolites (*m/z* < 650) commonly found in biological samples. The targeted peak picking extraction of MS^1^ data was performed by the *FullMS.R* function using 5 ppm and 0.005 Da as mass tolerance and *m/z* interval window as constraints, respectively, for general peak grouping and library interrogation. Ion polarity (neutral/negative/positive) and retention time are optional constraints that can be selected by users according to the customized library employed. The output generates a results list that includes the type of isotope or adduct annotated and the score that is reached by the peak shape based on the asymmetry factor (f) defined, as follows:

*f* = (t_Rf_ − t_Rmax_)/(t_Rmax_ − t_Ri_), (1)

where t_Rmax_ represents the retention time for the scan with the highest intensity at a given EIC and t_R_, t_Rf_ represents the retention time for the final scan, and t_Ri_ represents the retention time for the initial scan that together define the limits of the EIC. Therefore, *f* values that are closer to 1 indicate better peak symmetry. Where calculation of such factor was not feasible for chromatographic peaks below three scans across peak, and/or maximum signal intensity appeared as first or last scan (zero value in Tables).

Matched peaks were smoothed (cubic or smoothing spline) and evaluated regarding to their isotope peak intensity ratio (IPIR), peak-to-peak Pearson correlation (PPC), and peak-to-peak shape (PPS) ratios. The IPIR score was calculated according to the rule indicating that, in the absence of S or Br in the molecular formula, the ratio between monoisotopic and/or next isotopologues considered must be greater than one. Thus, IPIR was calculated, as follows:

(2) $$\mathrm{IPIR}=\frac{I_{k}}{I_{k+1}}$$

where *I*_k_ and *I*_k+1_ are the intensity of the monoisotopic peaks or the former and latter isotopologues.

The PPC score, based on Pearson correlation, was calculated with the following equation [22,23]:

(3) $$\mathrm{PPC}=\frac{\sum_{i=1}^{n}(I_{Pi}-\tilde{I_{P}})\left(I_{Fi}-\tilde{I_{F}}\right)}{\sqrt{\sum_{i=1}^{n}{\left(I_{Pi}-\tilde{I_{P}}\right)}^{2}}\sqrt{\sum_{i=1}^{n}{\left(I_{Fi}-\tilde{I_{F}}\right)}^{2}}}$$

where P and F are peaks “A” and “B”, *I*_Pi_ and *I*_Fi_ represents the intensity of a particular scan from a smoothed peak, $\tilde{I_{P}}$ and $\tilde{I_{F}}$ refer to the intensity sum for all scans forming the peak. The recommended cut-off value is PPC ≥ 0.7. 

Lastly, peak-to-peak shape (PPS) was defined as the ratio between the asymmetry factors from features within the same peak group (i.e. ions from the same metabolite), as follows [22,23]:

(4) $$PPS=\frac{f_{k1}}{f_{k2}}$$

where *f*_k1_ and *f*_k2_ are the asymmetry factors calculated with Equation (1) for a peak k1 and peak k2. Asymmetry factor ratios for features within the same peak group can be used as an indication of similarity due to the mandatory chromatographic elution behavior. Values of PPS below 0.3 and above 3 might reflect low similarity, in which case the metabolite with this considered precursor-product association should be discarded. IPIR, PPC, and PPS scores are implemented in the *ScoresMS1.R function* and graphical inspection of tentative assignments can be performed with the *plot_EIC.R* function.

Exploratory MS^1^ analysis was refined by R-MetaboList 2 through full-scan MS/MS data processing loading an upgraded AIF library (.csv format) that was previously released to study melanoma tissue and leukemia cell extracts while using a q-Orbitrap device [10]. In our case, MS/MS information of some underivatized metabolites commonly found in human urinary samples not considered in the aforementioned original library were additionally included (Table S4A for positive and S4B for negative ionization modes, respectively) following similar guidelines previously stated [10]. From these new urinary metabolites, accurate masses from fragments above 20% of relative abundance in the 0 to 30 eV CID range that is detailed in the mzCloud database populated the updated library used. Moreover, metabolites largely found in urine, cell, and medium samples with AIF fragments below *m/z <* 50 (i.e. urea and lactic acid) or assignments supported by only one ubiquitous ion (i.e. fragment at *m/z* 72.0444 from alanine) were discarded. Protocols to elaborate high-quality mass spectral libraries are described in the literature and can be readily used as an input for the MetaboList software [24]. AIF data analysis used 5 ppm and 0.08 min. as *m/z* and retention time tolerances as the main constraints, respectively, for proper peak alignment of precursors and their respective MS/MS fragments listed in Table S4A,B. Targeted data extraction was performed with a precursor-fragment ion mass-to-mass matching and having at least one fragment or all fragment ion (N) included in the MS/MS library matched as a minimum co-elution requirement. From assignments, the aligned EICs from MS^1^ and MS/MS were grouped and subsequently evaluated by the *ScoresDIA.R* function while using PPC, PPS, and product/precursor ion intensity ratio, with the last being defined as:

(5) $$F/PIon\;ratio=\frac{I_{max,F}}{I_{max,P}}$$

where *I*_max,F_ and *I*_max,P_ are the maximum EIC intensities corresponding to the fragment and precursor ion, respectively.

## 4. Conclusions

This study demonstrates the efficiency of R-MetaboList 2 for the simultaneous processing of multiplexed full-scan MS^1^ and MS/MS data from small molecule analysis. The complete flexibility of the methodology proposed facilitates the clear visualization and exhaustive quality assessment of findings from LC-HRMS data that were acquired by both q-TOF and q-Orbitrap devices analyzing underivatized human urine and myeloid leukemia cell and medium samples, respectively. Continuous upgradability of this strategy by users allows for the adaptation of a previously released in-house full-scan MS/MS q-Orbitrap library for R-MetaboList 2 analysis of data from both instrumental approaches considered. The flexibility of this approach permits the improvement of functions that were implemented in the previous R-MetaboList version as well as the incorporation of the new functions outlined above. Thus, detailed and accurate metabolite (mostly with *m/z* <250) profiling of samples was achieved, despite the complexity of merged full-scan analyses evaluated. Moreover, R-MetaboList 2 can facilitate quantitative studies and the election of the optimal collision energy for specific MS/MS fragments through the concurrent analysis of multiple fragmentation experiments. The proposed methodology represents a customizable and complementary alternative to the existing approaches to the automated processing of untargeted/targeted data dependent/independent MS/MS analyses, thus promoting global metabolomic strategies that are supported by recursive retrospective interrogation of multiplexed DIA data.

## Figures and Tables

**Table 1:** Statistical evaluation of peak groups for glutamine, phenylacetylglutamine, and phenylalanine performed by *ScoresDIA.R* function. Analysis performed on an LC-qTOF instrument. The recommended cut-off for PPC is ≥ 0.7. Good chromatographic similarity is indicated by PPS scores between 0.3 and 3. Abbreviations used: CE, Collision Energy; PPC, peak-to-peak Pearson correlation; PPS, peak-to-peak shape ratio.

	Experimental Fragment [M+H]^+^(*m/z*)	CE (eV)	PPC	PPS	^a^ Product/Precursor Ion Ratio
Glutamine					
	84.0444	5	0.93	0.22	0.23
	130.0499	5	0.97	1.00	0.72
	84.0444	10	0.87	0.67	0.74
	130.0499	10	0.87	0.67	0.74
	84.0444	20	0.87	0.67	0.99
Phenylacetylglutamine					
	84.0444	5	0.39	0.80	0.04
	130.0499	5	0.76	0.60	0.20
	84.0444	10	0.72	0.40	0.07
	130.0499	10	0.91	0.80	0.54
	136.0756	10	0.67	0.40	0.06
	147.0762	10	0.80	0.40	0.06
	84.0444	20	0.89	0.40	0.13
	130.0499	20	0.95	0.40	0.54
	136.0757	20	0.89	0.40	0.06
	147.0762	20	0.97	0.40	0.04
Phenylalanine					
	120.0809	5	0.60	3.00	0.77
	120.0809	10	0.87	1.50	1.39
	103.0543	20	0.76	2.00	0.43
	120.0809	20	0.93	1.50	1.05

^a^ Experimental intact mass of the precursor ion detailed in Table S4A (MS^1^ level).

**Table 2:** Statistical evaluation of glutathione, methionine, and tyrosine peak groups performed by *ScoresDIA.R* function. Extracted from data acquired on an LC-Q-Exactive Hybrid Quadrupole-Orbitrap device. Recommended values for PPC are ≥ 0.7. Good chromatographic similarity is indicated by 0.3 ≥ PPS ≥ 3. Abbreviations used: CE, Collision Energy; PPC, peak-to-peak Pearson correlation; PPS, peak-to-peak shape ratio.

	Experimental Fragment [M+H]^+^ (*m/z*)	CE (eV)	PPC	PPS	^a^ Product/Precursor Ion Ratio
Glutathione					
	76.0214	30	0.99	0.79	0.44
	116.0163	30	0.99	0.30	0.08
	144.0112	30	0.99	0.35	0.08
	162.0217	30	0.99	0.40	0.17
	179.0482	30	0.99	0.25	0.04
	233.0585	30	0.99	0.20	0.02
	130.0497	30	0.99	0.60	0.08
	84.0443	30	0.99	0.60	0.15
Methionine					
	133.0315	30	0.98	0.17	0.02
	104.0526	30	0.96	0.49	0.03
	61.0107	30	0.99	1.25	0.30
	56.0497	30	0.99	0.49	0.22
Tyrosine					
	147.0438	30	0.99	0.50	0.015
	136.0754	30	0.99	0.49	0.16
	123.0439	30	0.99	0.99	0.40
	119.0490	30	0.99	1.25	0.22
	95.0490	30	0.99	0.99	0.19
	91.0541	30	0.99	0.99	0.40

^a^ Experimental intact mass of the precursor ion detailed in Table S4A (MS^1^ level).

## Supplementary Materials

The following are available online at https://www.mdpi.com/2218-1989/9/9/187/s1, Figure S1. Output generated by the plot_EIC.R function for glutamine as detected by LC-qTOF. (A) Coelution profile for glutamine [M+H]+ and [M+NH4]+ adducts with a graphical abstract of the scores evaluated. Peak-to-peak Pearson correlation (PPC) was null whereas peak shape and mass accuracy acceptable for both peaks. (B) Extracted ion chromatogram (EIC) for the glutamine adducts found with dots indicating the scans forming the EIC and blue line the peak smoothed. (C) Quality control (QC) for the mass accuracy for each scan forming the EIC. Figure S2. Co-elution for extracted ion chromatograms for methionine precursor (blue line) and fragment at 133.0315 m/z (red line). The EICs show that these ions co-elute, indicating a low PPS (peak-to-peak shape ratio) due to the low intensity of the fragment ion and number of scans per peak. Table S1. Tentative assignments based on full-MS analysis in urine sample by LC-qTOF approach. Rt, retention time. Table S2. Tentative assignments based on full-MS analysis in cell sample by LC-Q-Exactive approach. Table S3. Tentative assignments based on full-MS analysis in medium sample by LC-Q-Exactive approach. Rt, retention time. Table S4. (A) In-house MS/MS library in positive ionization mode. (B) In-house MS/MS library in negative ionization mode. Table S5. Metabolites annotated by full-scan MS/MS of the urinary sample assayed with LC-qTOF device at CIDs of 5 eV. Rt, retention time. Table S6. Metabolites annotated by full-scan MS/MS of the urinary sample assayed with LC-qTOF device at CIDs of 10 eV. Rt, retention time. Table S7. Metabolites annotated by full-scan MS/MS of the urinary sample assayed with LC-qTOF device at CIDs of 20 eV. Rt, retention time. Table S8. Peak grouping for glutamine, phenylacetylglutamine and phenylalanine analyzed in urine by LC-qTOF at the three CE assayed. Rt, retention time; CE, collision energy. Table S9. Metabolites annotated by full-scan MS/MS analysis of the leukemia cell extract analyzed by q-Orbitrap. Rt, retention time. Table S10. Metabolites annotated by full-scan MS/MS analysis of the leukemia cell medium extract analyzed by q-Orbitrap. Three data sets converted to mzXML format for LC-qTOF and q-Exactive analysis of human urinary, leukemia cell and cell medium samples. Two R-scripts to reproduce results obtained in this research (AIF_Batch.R; Script_MetabolitesJournal.R).
