# Supplementary material for: R-MetaboList 2: A Flexible Tool for Metabolite Annotation from High-Resolution Data-Independent Acquisition Mass Spectrometry Analysis
Source: Metabolites. 2019 Sep 17;9(9):187. doi: 10.3390/metabo9090187 (PMC6780920; doi:10.3390/metabo9090187)
Supplement: Supplementary file 1 [file metabolites-09-00187-s001.zip › Supplementary Materials/Supplementary Tables/Table S8_OKdef.docx]

**Table S8**. Peak grouping for glutamine, phenylacetylglutamine and phenylalanine found in the urinary sample analyzed by full-scan MS^1^ and MS/MS LC-qTOF.

| Metabolite | [M+H]^+^ (m/z) | Rt (min) | Peak width (min) | Peak area | ^a^Peak Assymetry | Signal Intensity | Mass shift (ppm) | ^b^CE (eV) |
| --- | --- | --- | --- | --- | --- | --- | --- | --- |
| Glutamine | 147,0764 | 1,36 | 0,09 | 45613 | 1,5 | 11511 | 0,19 | 0 |
| Glutamine | 84,0443 | 1,38 | 0,07 | 11447 | 0,3 | 2644 | -0,74 | 5 |
| Glutamine | 130,0499 | 1,37 | 0,09 | 34151 | 1,5 | 8267 | 0,15 | 5 |
| Glutamine | 84,0445 | 1,37 | 0,10 | 39140 | 1,0 | 8557 | 0,65 | 10 |
| Glutamine | 130,0500 | 1,37 | 0,10 | 36692 | 1,0 | 8509 | 0,40 | 10 |
| Glutamine | 84,0445 | 1,38 | 0,10 | 53014 | 1,0 | 11478 | 1,06 | 20 |
| Phenylacetylglutamine | 265,1184 | 6,62 | 0,12 | 229958 | 2,5 | 69029 | 0,6 | 0 |
| Phenylacetylglutamine | 84,0446 | 6,63 | 0,05 | 7837 | 2,0 | 2508 | 1,8 | 5 |
| Phenylacetylglutamine | 130,0499 | 6,63 | 0,09 | 43928 | 1,5 | 13893 | 0,5 | 5 |
| Phenylacetylglutamine | 136,0758 | 6,63 | 0,00 | 1076 | 0.0 | 1076 | 0,6 | 5 |
| Phenylacetylglutamine | 147,0765 | 6,63 | 0,00 | 1155 | 0.0 | 1155 | 0,6 | 5 |
| Phenylacetylglutamine | 84,0444 | 6,63 | 0,07 | 15160 | 1,0 | 4837 | 0,1 | 10 |
| Phenylacetylglutamine | 130,0500 | 6,63 | 0,10 | 120687 | 2,0 | 36871 | 0,7 | 10 |
| Phenylacetylglutamine | 136,0756 | 6,63 | 0,07 | 14442 | 1,0 | 4374 | -0,3 | 10 |
| Phenylacetylglutamine | 147,0763 | 6,63 | 0,07 | 14736 | 1,0 | 4729 | -0,9 | 10 |
| Phenylacetylglutamine | 84,0444 | 6,64 | 0,07 | 27477 | 1,0 | 8768 | 0,6 | 20 |
| Phenylacetylglutamine | 130,0499 | 6,64 | 0,10 | 123101 | 1,0 | 37560 | 0,3 | 20 |
| Phenylacetylglutamine | 136,0757 | 6,64 | 0,07 | 12589 | 1,0 | 4341 | 0,3 | 20 |
| Phenylacetylglutamine | 147,0763 | 6,64 | 0,03 | 7085 | 1,0 | 2793 | -0,7 | 20 |
| Phenylalanine | 166,0862 | 5,54 | 0,069 | 25731 | 1,0 | 7988 | -0,71 | 0 |
| Phenylalanine | 120,0809 | 5,53 | 0,069 | 20109 | 3,0 | 6165 | 2,01 | 5 |
| Phenylalanine | 120,0808 | 5,53 | 0,086 | 37137 | 1,5 | 11088 | 1,24 | 10 |
| Phenylalanine | 103,0543 | 5,54 | 0,052 | 10056 | 2,0 | 3452 | 1,74 | 20 |
| Phenylalanine | 120,0808 | 5,54 | 0,086 | 26819 | 1,5 | 8408 | 1,04 | 20 |

^a^Zero value for chromatographic peaks below three scans across peak and/or maximum signal intensity at first or last scan.

^b^CE: collision energy assayed (CID).
