# Supplementary figures and images for: R-MetaboList 2: A Flexible Tool for Metabolite Annotation from High-Resolution Data-Independent Acquisition Mass Spectrometry Analysis
Source: Metabolites. 2019 Sep 17;9(9):187. doi: 10.3390/metabo9090187 (PMC6780920; doi:10.3390/metabo9090187)

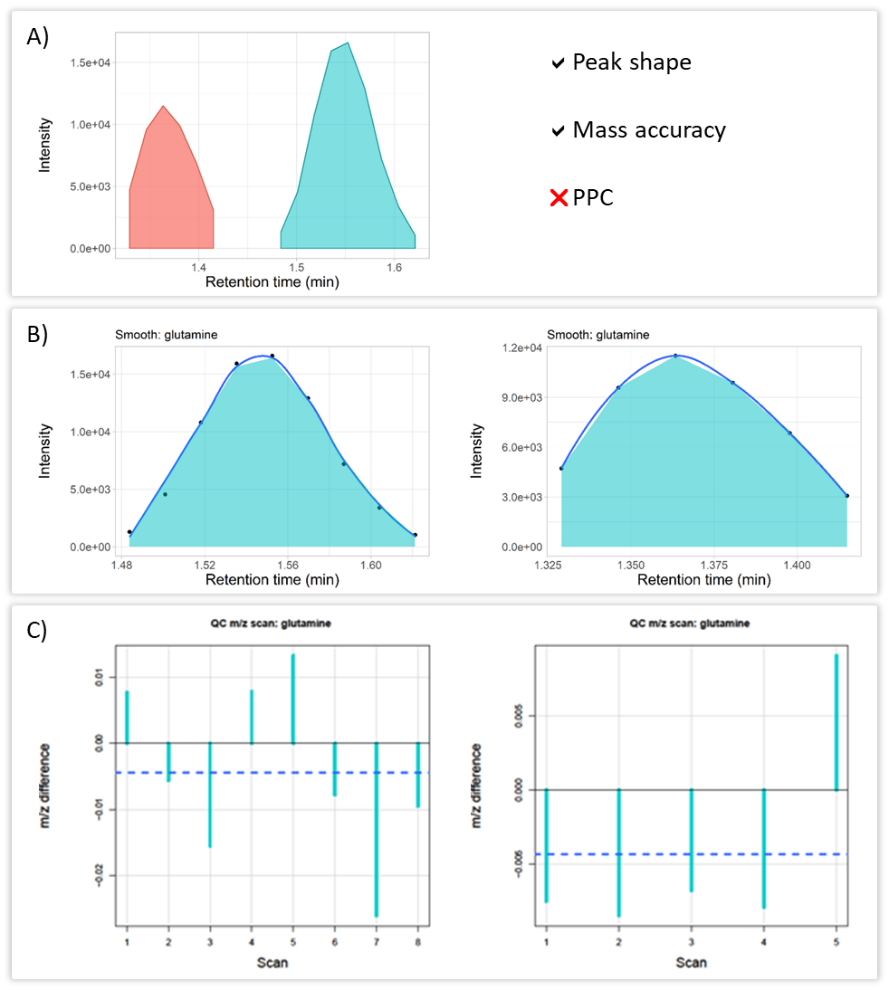

Supplement: Supplementary file 1 [file metabolites-09-00187-s001.zip › Supplementary Materials/Supplementary Figures/Figure S1.png]

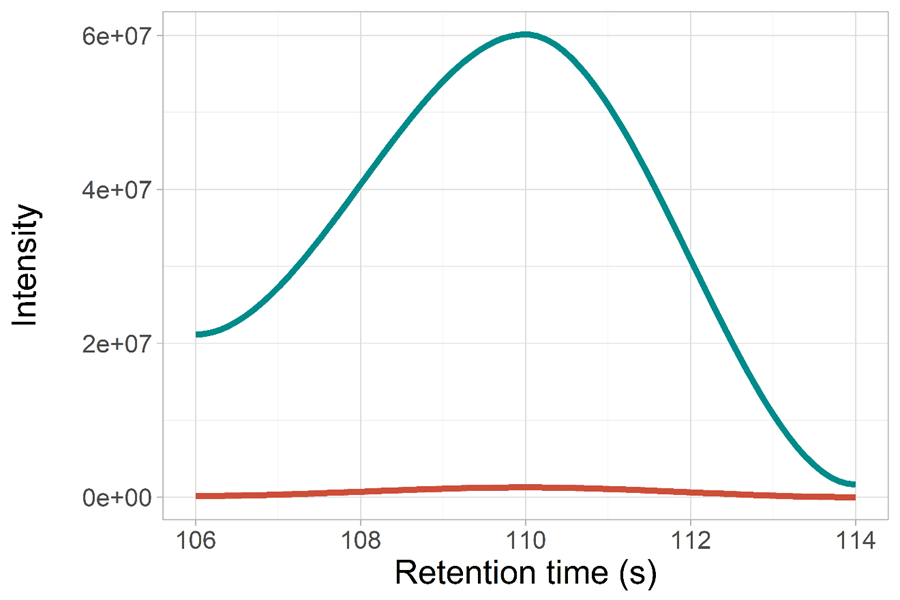

Supplement: Supplementary file 1 [file metabolites-09-00187-s001.zip › Supplementary Materials/Supplementary Figures/Figure S2.png]
